# Mixed-methods study to assess delay among patients with tuberculosis in an urban setting of Bangladesh

**DOI:** 10.1371/journal.pone.0327348

**Published:** 2025-07-01

**Authors:** Shahriar Ahmed, Samanta Biswas, Tanjina Rahman, Ahammad Shafiq Sikder Adel, S. M. Zafor Shafique, Refah Tamanna, Kamal Ibne Amin Chowdhury, Sayera Banu

**Affiliations:** 1 International Centre for Diarrhoeal Disease Research, Bangladesh (icddr, b), Dhaka, Bangladesh; 2 Rollins School of Public Health, Emory University, Atlanta, Georgia, United States of America; Bangabandhu Sheikh Mujib Medical University (BSMMU), BANGLADESH

## Abstract

**Background:**

Tuberculosis (TB) regained its position as the leading cause of death globally from a single infectious disease agent in 2024. Delayed diagnosis and treatment hamper effective TB control. We investigated the duration of diagnostic and treatment delay along with the associated factors among people with pulmonary TB in Bangladesh.

**Methods:**

A mixed-method study was conducted between December’19 and March’21, at icddr,b TB Screening and Treatment Centres (TBSTCs), Dhaka. We interviewed people with TB (PWTB) seeking care at these TBSTCs using a structured questionnaire to collect data on socio-demographic, clinical and healthcare seeking behaviors. We used established frameworks to define stages of delay and associated factors. Qualitative interviews were conducted among a subset of participants to gain further insight into the factors associated with delay.

**Results:**

We enrolled 895 PWTB with mean (±SD) age 36.6 (±16.1) years; majority of participants were males (69.9%) and living in urban areas (82.3%). The median (IQR) patient delay estimated was 47 (29–72) days, with diagnostic delay 45 (30–70) days and treatment delay 2 (2–4) days. The predictors of delay were those with diabetes (OR 2.0, 95% CI – 1.11, 3.42), who initially self-treated (OR 2.1, 95% CI – 1.09, 3.88), and were bacteriologically diagnosed (OR 3.7, 95% CI – 1.31, 10.46). Qualitative approach supported the quantitative findings and revealed the practice of visiting formal physicians during worsening illness, neglecting to acknowledge signs or symptoms consistent with TB, lack of TB related knowledge, and financial insolvency as major reasons for delay.

**Conclusion:**

Our findings showed that improper health-seeking behavior is one of the major drivers of patient delay. Thus, targeted programmatic intervention to raise community awareness on TB and its care services with a special focus on informal providers can help reduce this delay.

## Introduction

Tuberculosis (TB) remains a global concern as only 75.9% of the estimated 10.8 million TB cases were notified in 2023, with 1.25 million deaths [1]. Underreporting and underdiagnosis are the main causes of the discrepancy between the estimated and reported numbers. During 2023, more than 20% of the estimated number of people with TB (PWTB) were missed in Bangladesh and estimated number of deaths from TB was approximately 44,000 [1].

Prompt diagnosis and successful treatment of PWTB can prevent millions of deaths globally every year. Yet, this persistent gap of undetected and untreated cases remains a threat. Major challenges in closing this gap include delay in diagnosis and initiating treatment for TB after onset of symptoms. Patient delay, that includes diagnostic delay and/or treatment delay after diagnosis, results in increased community transmission, progression in disease severity and a higher risk of mortality [2,3]. This delay in diagnosis results from both delay in care seeking by patients, as well as delays by the healthcare system in reaching a final diagnosis [4]. In low and middle income countries (LMICs), evidence suggests that more than 42% people sought care after a delay of a month or more [5]. Living in rural areas, poor knowledge, and treatment seeking from informal healthcare providers have been associated with increased delay [6] elsewhere. Relevant studies are infrequent from Bangladesh, and resulting lack of data is a major barrier for policymakers in developing effective strategies aimed at mitigating this issue.

In the country, icddr,b is providing TB diagnostic and treatment services through TB Screening and Treatment Centres (TBSTC) for more than a decade [7]. Leveraging this platform, we planned to estimate the delay in diagnosis and treatment initiation along with the related factors by interviewing persons seeking care from these TBSTCs and further corroborating these findings with in-depth interviews (IDIs). This study aimed to gather evidence on the existing challenges in the TB care cascade, which policymakers can use to develop strategies to address these gaps.

## Methods

### Study design

We conducted this mixed-method cross-sectional study at the seven icddr,b TBSTCs located in Dhaka city, between December 2019 and March 2021. These TBSTCs are located in places suitable to facilitate easy access for patients seeking healthcare in the private sector. People with presumptive TB are referred to these TBSTCs for Xpert testing and chest X-ray by private physicians, pharmacies, NGO partners along with many private and public healthcare facilities. To provide context for the quantitative data and capture various patient perspectives on the delays, qualitative methods were employed.

The study was part of a bigger umbrella project at icddr,b titled USAID’s Research for Decision Makers (RDM) activity and detailed report of the project is available online [8]. The report outlined specific procedures for selecting a representative sample, administering structured surveys, and employing statistical techniques for quantitative data analysis, ensuring the reliability and validity of the results.

### Study population

All PWTBs diagnosed at the TBSTCs, as well as those diagnosed elsewhere who visited the TBSTCs for treatment and met the study criteria were approached and enrolled upon informed written consent from December 14, 2019 to September 5, 2020.

For the in-depth interviews, individuals were chosen purposively using a method that combined their sociodemographic information with relevant findings from the quantitative assessment. The process was intended to ensure that different types of experiences and folks from varied backgrounds were represented. Key variables related to individuals’ backgrounds included age, gender, education, socioeconomic position, type of work, members in the household and where they live.

Along with demographic information, numbers from the quantitative assessment guided the method of selecting participants in the qualitative arm of the study. The date samples were sent in for the GeneXpert test, the date the results were collected and the date a patient started DOT were included, since we believed these events could cause a delay in diagnosis and treatment. As lockdown during the recent pandemic had an impact on healthcare seeking behavior (HSB), we also considered this during selection of participants.

Due to the pandemic, the first communication with chosen participants was done by telephone. The timing and place for interviews was decided in consultation with the participants prioritizing their convenience. Both the participants and the interviewers maintained a safe distance and used personal protective equipment during interviews that were held in the participants’ homes.

### Sample size

The calculated sample size was 710 PWTB based on mean (±SD) patient delay of 54 ± 28 days, utilizing 4% relative precision with 95% confidence interval (CI), and 10% non-response rate [9]. We enrolled 895 PWTB for quantitative interviews and conducted 21 IDIs, till data saturation was reached.

### Data variables

The quantitative interviews were conducted using structured questionnaires and data were digitally entered directly into an online database using tabs. Entered data was checked weekly by data management personnel to ensure completeness and accuracy. The primary outcome variable was patient delay which included diagnostic and treatment delay. Diagnostic delay was considered from onset of symptom to date of confirmed diagnosis, while treatment delay was considered from date of confirmed diagnosis to DOTS enrolment [3,10]. Existing literature suggest that a sick person usually seeks treatment within two to three weeks of experiencing symptom. However, we added an additional 7 days and set the cut-off at 30 days to determine the patient delay which align with findings of most studies [10,11].

We considered Anderson and Newman’s framework of health services utilization to select the demographic covariates that are associated with patient delay [12,13]. This framework consists of three individual determinants- i. Predisposing, ii. Enabling, and iii. Illness level. We adopted age, gender as demographic and education, area of dwelling, marital status as well as family housing characteristics as social structure from predisposing factors. In addition, we incorporated the Thaddeus and Maine (1994) framework, which, although originally developed to understand delays in maternal health care, has since been widely used for TB and other diseases where timely care-seeking is critical [14,15]. This model delineates three stages of delay: (i) delay in the decision to seek care (patient delay), (ii) delay in reaching a healthcare facility (access delay), and (iii) delay in receiving appropriate care (diagnostic and treatment delay). Based on this structure, we categorized TB knowledge, perceived stigma, health beliefs, and perceived severity of illness as factors influencing the decision to seek care. Travel distance, transportation availability, duration of sufferings, healthcare costs, and household decision-making dynamics were included as access-related barriers.

Regarding age and education, we used completed years. Age was categorized into seven different groups such as 5–14, 15–24, 25–34, 35–44, 45–54, 55–64, and greater than or equal to 65 years. Similarly, educational attainment was categorized into seven groups: 0 years (unable to read and write), 1–5 years (primary), 6–8 years (junior secondary), 9–10 years (secondary), 11–12 years (higher secondary), 13–16 years (undergraduate), and ≥17 years (postgraduate and above). For symptom assessment, participants were asked whether they were currently experiencing any TB-related symptoms. Additionally, we collected information on whether the participant had been in close contact with a person having active TB, or had a family member diagnosed with TB in the past four months, to assess recent TB exposure. Information on socio-demographics, clinical information, knowledge and awareness of TB, care seeking behavior, perceived causes of delay, and barriers to accessing healthcare was collected. The IDIs provided insight into the perspectives of those with lived experience regarding the overall issues and obstacles encountered during diagnosis and initiation of treatment.

### Data analysis

Data were analysed by using statistical software Stata/SE version 15. Frequency, proportions, mean (±SD) and median (IQR), were reported to describe socio-demographic characteristics, symptom profile, TB knowledge and healthcare seeking behaviour (HSB). The wealth index measures a household’s overall living standard and was derived as a part of participants’ socio-economic characteristics utilizing principal component analysis [16]. Households were scored based on characteristics yielding five quintiles – poorest, poor, middle, rich and richest. Knowledge score of the participants was obtained as a composite value by assigning ‘1’ for correct response and ‘0’ for incorrect. Total score of 50 was divided into percentile to indicate different knowledge level: adequate (76%−100%), moderate (51%–75%) and inadequate (≤50%). Logistic regression (unadjusted) was performed to examine relationship between delay and socio-demographics, clinical factors, TB related knowledge, and HSB to find predictors of delay. Statistical significance was considered at p-value<0.05. We assessed the assumption of linearity between the continuous predictor variables and the outcome, and observed a non-linear association. As a result, we transformed the covariate into categories as age and educational attainment were categorized for analysis. To evaluate the relationship between patient delay and various explanatory factors, both unadjusted and adjusted odds ratios were estimated using binary logistic regression models. The unadjusted associations were first explored through bivariate logistic regression, presenting crude odds ratios (COR) with 95% confidence intervals (CI). The covariates included in the analysis were age, educational attainment, marital status, household wealth index, place of residence, presence of TB-related symptoms, and history of contact with TB cases. Variables found to be significantly associated with the patient delay at a p-value threshold of <0.05 in the bivariate analysis were subsequently included in the multivariable logistic regression model to identify the predictors of patient delay. After adjusting for the statistically significant covariates, multivariate logistic regression was used to identify the variables associated with patient delay. Considering two-tailed tests of significance, odds ratio (OR) calculated at 95% CI. Also, data were analysed by observing the effect of COVID-19 on HSB of PWTB before, during and after the lockdown in Bangladesh.

We employed a thematic analysis approach to explore in-depth perspectives and contextual factors influencing HSB and patient delay. All audio-recorded interviews were transcribed verbatim and reviewed by two anthropologists to ensure consistency. Transcripts were read multiple times to ensure familiarity, followed by systematic coding. Two qualitative researchers came up with a coding framework by incorporating both deductive codes drawn from the interview guide and inductive codes that emerged from the data itself. The coded data were then organized into themes through a repeated process of comparison and categorization.

To ensure inter-coder reliability, two coders analyzed the data independently, and discrepancies in coding were resolved through systematic discussions. Conceptual memos and codebooks were used to document analytic decisions and theme development. Triangulation was performed by cross-verifying information from different sources, including interviews and field notes, to enhance credibility. In addition, the qualitative team considered participant checking by sharing preliminary findings with selected participants to validate interpretations and ensure alignment with their intended meanings. This rigorous process allowed for the development of rich, contextually grounded themes that complemented and expanded upon the quantitative findings.

### Ethical approval

The study protocol (PR-19104) was approved by the Institutional Review Board of icddr,b. From all participants aged ≥18 years, informed written consent was obtained and assent was obtained from guardians of children (aged 11–17 years) who consented to participate.

## Results

In quantitative assessment, among the participants (n = 895), majority were male (69.9%), residing in urban settings (82.3%) presenting with cardinal symptoms of cough >2 weeks (83.3%), significant weight loss (83.6%), fever (84.0%) and night sweats (60.1%). Their mean (±SD) of age was 36.6 (±16.1) years, and BMI was 20.1 (±3.8) kg/m^2^. A small percentage of participants was found to be unable to read and write (13.5%). The family wealth index of participants showed a relatively uniform distribution of income levels with the majority falling within the poorest (20%) and poor (21.8%) wealth quintiles (**Table 1**).

**Table 1:** Demographic and clinical profiles of study participants (n = 895) enrolled in icddr,b TB Screening and Treatment Centres in Dhaka, Bangladesh during December’19 to March’21.

Characteristics		Total (N = 895)	
		**N**	**(%)**
Age (in years)	5–14	16	(1.7)
	15–24	257	(28.7)
	25–34	173	(19.3)
	35–44	151	(16.9)
	45–54	137	(15.3)
	55–64	103	(11.5)
	65+	58	(6.5)
Gender	Male	626	(69.9)
	Female	269	(30.1)
Educational status	Unable to read and write	121	(13.5)
	Primary (1–5)	249	(27.8)
	Junior secondary (6–8)	146	(16.3)
	Secondary (9–10)	151	(16.9)
	Higher secondary (11–12)	108	(12.1)
	Undergrad (13–16)	83	(9.3)
	Postgrad and above (>=17)	37	(4.1)
Marital status	Married	873	(97.5)
	Others	22	(2.5)
Living area	Urban	737	(82.3)
	Rural	158	(17.7)
BMI according to the World Health Organisation’s Asian classification	Underweight (<18.5 kg/m^2^)	317	(35.4)
	Normal (18.5–22.9 kg/m^2^)	397	(44.4)
	Overweight (23.0–24.9 kg/m^2^)	89	(9.9)
	Obese (≥25.0 kg/m^2^)	92	(10.3)
TB symptoms	Cough	890	(99.4)
	Fever	752	(84.0)
	Significant weight loss	748	(83.6)
	Fatigue	782	(87.4)
	Loss of appetite	726	(81.1)
	Night sweats	538	(60.1)
	Shortness of breath	274	(30.6)
	Wheezing	191	(21.3)
	Haemoptysis	180	(21.1)
Diabetes		202	(22.6)
Hypertension		83	(9.3)
TB case type	New case	791	(88.4)
	Re-treatment case	104	(11.6)
History of TB contact		33	(3.7)
TB diagnosis	Bacteriologically confirmed	868	(97)
	Clinically diagnosed	27	(3.0)

All IDI participants (n = 21) were bacteriologically confirmed cases, of whom only 5/21 were new cases, majority were male (13/21) and had mean (±SD) age of 37.2 (±14.9) years. Most participants (15/21) passed Class 5–10 or below education. Over half (12/21) were service-holders, followed by businessmen, garment workers, farmers or day labourers.

### Knowledge and awareness related to TB

Most of the participants knew about TB (850, 95%), mostly through their friends and family (61.1%) and advertisements (60.9%). In addition, most were aware of the symptoms before being diagnosed (86.2%). Two-third participants (62.1%) knew that TB could be acquired at any time although some (16%) were not aware that TB might be spread by sneezing, coughing, and shouting. Some of the participants also had the misconception that TB occurred genetically (11.8%) (**Table 2).** Majority were unaware of the various types of TB (87.9%) although many recognized that TB affects the lungs (79.3%), while others noted its impact on abdomen (11.1%) or any part of the body (10.4%). Two-third participants believed that anyone exposed to TB would become ill (60.2%). Majority believed that TB can be fully cured (92.9%) and one could have TB again if they did not complete the anti-TB treatment course (88.9%). Most stated knowing where to go if they thought they might have TB (96.1%), majority mentioned government health facilities (74.9%), and some mentioned Non-government organization (NGO) clinics (10.7%) as places they can seek care from. Nearly two-third participants perceived TB to be a highly serious issue (61.4%). Most participants accurately reported that TB treatment is provided free of charge (83.7%) (**Table 3**). The mean (±SD) of TB knowledge score (n = 850) was calculated 32.2 (±7.1), which revealed around two-thirds had a moderate level (63.7%) of knowledge.

**Table 2:** Knowledge of TB among study participants (n = 850) enrolled in icddr,b TB Screening and Treatment Centres in Dhaka, Bangladesh during December’19 to March’21.

		Total (N = 850)	
		**N**	**(%)**
Commonly known TB symptoms:*			
	Cough > two weeks	711	(97)
	Fever	526	(71.8)
	Haemoptysis	313	(42.7)
	Chest pain	305	(41.6)
	Fatigue/anorexia	256	(34.9)
	Unexplained weight loss	254	(34.7)
	Evening rise of temperature	254	(34.7)
	Respiratory distress	183	(25)
	Night sweats	174	(23.7)
	Headache	107	(14.6)
	Numbness/tingling	102	(13.9)
	Nausea/vomiting	74	(10.1)
	Others (Diarrhoea/constipation, gibbous, joint pain etc.)	29	(4.0)
Source of information on TB:*			
	Family/friends	519	(61.1)
	Advertisements	518	(60.9)
	Doctor	292	(34.4)
	Billboards	258	(30.4)
	Cured TB patient	200	(23.5)
	Health worker	182	(21.4)
	Newspapers	158	(18.6)
	Radio	133	(15.7)
	Non-government organisation (NGO)	84	(9.9)
	Others (including in schools)	91	(10.8)
One can get TB by:*			
	Coughing, sneezing, shouting	697	(82)
	Quick causal contact	346	(40.7)
	Sharing utensils/food/drinks	280	(32.9)
	Smoking cigarettes, biddi or tobacco	264	(31.1)
	Exchanging saliva or other bodily fluids	219	(25.8)
	Blood transmission	85	(10)
	Others (air, genetic, dust, injection, low immunity, changes in weather)	71	(8.4)
	Don’t know	59	(6.9)
Patients with TB may prevent spreading it to others by:*			
	Wearing a mask	666	(78.4)
	Covering their mouth and nose when coughing or sneezing	447	(52.6)
	Avoiding sharing utensils	398	(46.8)
	Avoiding enclosed spaces when there are other people there	293	(34.5)
	Avoiding shaking hands	230	(27.1)
	Avoiding sleeping in the same room as other people	149	(17.5)
	Not touching items in public places	90	(10.6)
	Others (stopping smoking, praying, having good nutrition etc.)	160	(18.9)
People affected by TB:*			
	Men	701	(82.5)
	Women	661	(77.8)
	Elderly (>64 years)	599	(70.5)
	Adults (15–64 years)	592	(69.7)
	Children (<15 years)	583	(68.6)
	Don’t know	100	(11.8)

* Multiple responses allowed

**Table 3:** Perception of TB treatment among study participants (n = 850) enrolled in icddr,b TB Screening and Treatment Centres in Dhaka, Bangladesh during December’19 to March’21.

Perception of TB		Total (N = 850)	
		N	(%)
**Perceived benefits from starting treatment early and finishing the full course:**			
	Early cure	682	(80.2)
	Less transmission	291	(34.2)
	Fewer side effects	241	(28.4)
	Shorter treatment time	204	(24.0)
	Fewer complications	130	(15.3)
	Less costly	118	(13.9)
	Lower risk of drug resistance	84	(9.9)
	Others (patient can do everything they could before, strength for work, make a better life)	3	(0.4)
	Don’t know	66	(7.8)
**Perceived time taken to cure TB:**			
	6 months	657	(77.3)
	12 + months	8	(0.9)
	When the doctor tells me it is cured	6	(0.7)
	When I feel completely better	5	(0.6)
	Don’t know	174	(20.5)
**They heard about where to go from:**			
	Family/friends	483	(56.8)
	Advertisements	386	(45.4)
	Health worker	231	(27.2)
	Cured TB patient	215	(25.3)
	Billboard/signboard	209	(24.6)
	Radio	127	(14.9)
	Newspaper	126	(14.8)
	NGO	95	(11.2)
	Doctor	74	(8.7)
	School	53	(6.2)
	Others (self-known, word of mouth, history of TB)	16	(1.9)
	Don’t know where to go	13	(1.5)

* Multiple responses allowed

During qualitative interviews, most of the participants (17/21) were found to possess basic knowledge of TB. Among them, four participants (4/17) perceived TB to be transmitted through coughs, sneezing and sharing food with confirmed TB patients.

A 35-year-old male participant described,

*“I had been having [a] cough for at least two–three months, but it did not contain any sputum or blood. I thought the cough might have been caused by dust.”*

### Healthcare-seeking behaviour

The median (IQR) duration between seeking treatment and any symptom onset was 45 (30–65) days and median (IQR) cough duration was 30 (20–60) days as stated by study participants. Two-fifths reported pharmacies nearby (42.1%) or private healthcare facilities (40.9%) as their first point of care seeking while majority (92.2%) reported they only sought care when their condition was getting worse. The median (IQR) time between confirmed diagnosis and first care-seeking was 28 (15–55) days. More than two-third participants (71.7%) had reported a delay in care seeking and finally diagnosis – mostly because they expected their symptoms to resolve on their own (82.2%) but also because of self-medication (27.4%), financial hardship (21.8%), misdiagnosis (19.3%), seeking help from the traditional healers (3.3%), stigma (1.2%) and others (4.5%).

In qualitative exploration, majority (12/21) reported that they typically visited local pharmacies first for symptoms such as cough, fever and chest pain. A few participants received their first medical care from a quack physician or spiritual healer (huzur). Most of them (19/21) expressed that they sought medical care from formal physicians while deteriorating their health condition.

A 50-year-old male participant stated,

*‘I started taking medicine from [the] pharmacy after 7 or 15 days of getting [a] fever and cough. But the medicine was not working; then, I went to the hospital’*

Participants also mentioned financial insolvency as a reason of their delay to seek healthcare, especially from formal providers.

A 56-year-old male participant mentioned,

*“Due to my insolvency, I have been suffering from this sickness for two months. Many people, including my wife and son, asked me to visit a doctor. If I would have had money, I wouldn’t procrastinate the treatment for two months.*

Participants also mentioned their negligence, lack of knowledge about TB, prioritising job and other activities played significant role for their treatment delay.

### Access to health facilities

Majority reported the mean (±SD) distance of their nearest health facility (96.1%) and their nearest DOTS centre (90.9%) from their home was 2.4 (±2.4) km, and 2.5 (±2.3) km respectively. It took 20.3 (±14.6) minutes to travel to their nearest healthcare facility (96.3%), and 21.4 (±13.8) minutes to travel to the nearest DOTS facility (91%). Only a few (2.7%) reported difficulties visiting DOTS centres for medication due to a long distance (53.8%), time constraints (19.2%), financial problems (15.4%) or feeling too weak to travel (11.5%). Moreover, 29.8% reported that their median (IQR) monthly pone.0327348 wage loss would be Tk. 1,800 (1200–3500) if they had to visit the DOTS facility for daily medication, 39.7% reported no wage loss.

### Delay in diagnosis and treatment initiation

**Fig 1:**
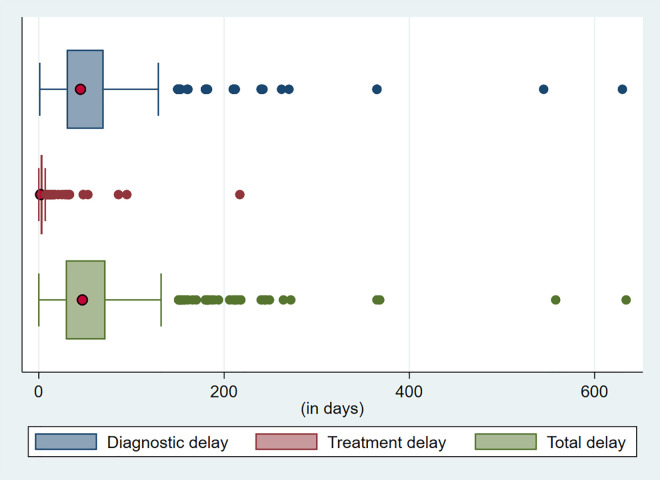
Delay in diagnosis and treatment initiation of study participants’ (n = 895) enrolled in icddr,b TB Screening and Treatment Centres in Dhaka, Bangladesh during December’19 to March’21.

Median (IQR) diagnostic delay (n = 867) was 45 (30–70) days, while the median (IQR) treatment delay (n = 883) was found to be 2 (2–4) days. Thus, reported median (IQR) total delay was 47 (29–72) days (**Figure 1**). Accordingly, over two-thirds (73.6%) were found to have delayed in seeking treatment. Almost all indicators were crudely significant with delay (**Table 4).** After adjusting covariates, the likelihood of delay for diabetics were two times higher than non-diabetics; among bacteriologically diagnosed were 3.7 times higher than those clinically diagnosed, and those who had self-treated were twice as likely to delay than those who did not.

**Table 4:** Univariate and multivariate logistic regression for measuring the predictors of delay among study participants (n = 850) enrolled in icddr,b TB Screening and Treatment Centres in Dhaka, Bangladesh during December’19 to March’21.

Covariates	Crude OR (95% CI)	Adjusted OR (95% CI)
Age in years	1.01 (1–1.02)*	1.0 (0.98–1.01)
Cough duration in days	1.02 (1.02–1.03)*	1.0 (1–1.01)
Cough >2 weeks (Reference: ≤ 2 weeks)	3.37 (2.34–4.86)*	2.44 (1.39–4.27)*
Night sweats (Reference: no)	0.67 (0.49–0.92)*	0.55 (0.34–0.84)*
Wheezing (Reference: no)	1.58 (1.07–2.33)*	1.25 (0.75–2.09)
Diabetes (Reference: no)	1.73 (1.17–2.55)*	1.95 (1.11–3.42)*
Education (Reference: unable to read and write)		
Primary	0.94 (0.55–1.61)	1.16 (0.56–2.4)
Junior secondary	0.76 (0.42–1.36)	0.94 (0.41–2.13)
Secondary	0.58 (0.33–1.03)	0.78 (0.35–1.76)
Higher secondary	0.48 (0.26–0.86)*	0.44 (0.19–1.05)
Undergrad	0.42 (0.22–0.78)*	0.56 (0.23–1.38)
Postgrad and above	0.59 (0.25–1.35)	0.58 (0.17–1.93)
Marital status (Reference: single)		
Married	1.66 (1.22–2.27)*	1.35 (0.77–2.36)
Area of living (Reference: rural)	0.52 (0.33–0.81)*	0.61 (0.33–1.15)
Patients knew about TB symptoms before being diagnosed (Reference: did not know)	1.06 (1.01–1.1)*	1.03 (0.95–1.11)
TB can affect people of any age (Reference: no)	1.08 (1.01–1.16)*	0.99 (0.88–1.11)
First visit before confirmed TB diagnosis (Reference: government health facilities)		
NGO/NGO clinic	1.15 (0.47–2.83)	2.49 (0.79–7.85)
Nearby pharmacy	1.98 (1.25–3.13)*	1.51 (0.78–2.92)
Private health facilities	0.96 (0.62–1.49)	1.78 (0.94–3.37)
Time between first visit and confirmed diagnosis	1.1 (1.08–1.12)*	1.09 (1.07–1.11)*
Type of TB patient (Reference: pulmonary CD)	3.13 (1.45–6.77)*	3.7 (1.31–10.46)*
Delay in diagnosis (Reference: no)	6.48 (4.67–8.99)*	1.7 (0.76–3.80)
Hoped symptoms would go away	3.66 (2.68–5)*	1.19 (0.57–2.49)
Shortage of money	2.28 (1.40–3.72)*	0.94 (0.49–1.82)
Self-treatment	2.69 (1.70–4.26)*	2.05 (1.09–3.88)*
Misdiagnosis	3.48 (1.92–6.3)*	2.03 (0.93–4.42)

* refers to significance considering p < 0.05

During the qualitative interviews, most of the participants (18/21) perceived doctor to be responsible for their delayed diagnosis as they experienced wrong treatment on their first visit.

A 51-year-old female participant mentioned,

*‘No remarks on the doctors’ failure! They could not diagnose the disease in spite of having such valuable degrees. We suffered and wasted money’.*

Participants also mentioned that the delay in collecting reports influenced their treatment initiation on time. They also mentioned some challenges during treatment initiation exacerbating the situation such as living outside of Dhaka, remote residence, lack of alternative support/family member to collect medicine.

### Effect of COVID-19

The COVID-19 emergency situation was declared during study period and the associated challenges significantly decreased study enrolments. Most participants (71.7%) were enrolled prior to the nationwide lockdown (14 December 2019–24 March 2020), a smaller proportion (4.9%) during the lockdown (25 March – 30 May 2020) and the remaining (23.4%) were enrolled after the lockdown (31 May – 5 September 2020). The median (IQR) length of suffering (before seeking healthcare) was 45 (30–65) days pre-COVID and 37.5 (27.5–70) days during lockdown. The median (IQR) duration of suffering dramatically increased to 60 days (30–90) after lockdown. Majority of participants sought care after worsening of illness, particularly after lockdown (96.7%, p < 0.01). Notably before COVID, participants initially visited nearby pharmacies (42.5%), then private healthcare facilities (38.9%), which reversed (pharmacies: 18.1%; private healthcare facilities: 70.5%) significantly during lockdown (p < 0.01), and returned to the initial status post-lockdown (45.9% and 40.7% respectively). Proportion of females seeking healthcare surged significantly during the strict lockdown (47.7%, p < 0.01) period than pre-COVID (27.4%) or post-lockdown (34.5%) periods.

## Discussion

Our study enrolled participants with TB, referred mostly from private providers of Dhaka, and found median (IQR) patient delay of 47 (29–72) days. Nearly three-fourths (659, 73.6%) of the participants had delayed seeking healthcare. This was consistent with the median total delay of 35–97 days found in previous studies from Bangladesh and other Asian countries [17–19]. Similar to our study, which found median (IQR) diagnostic delay of 45 (30–70) days and a rather smaller treatment delay of 2 (2–4) days, most studies reported a much lengthier delay in diagnosis than in treatment initiation. Many studies from Asia and Africa, including systematic reviews and meta-analyses showed a diagnostic delay of as high as 366.5 days with a median of 23–91 days [17–20]. We found a minimal treatment delay of 2 (2–4) days, which is also consistent with multiple studies which reported 1–8 days [17–20].

These variations in delay may be attributed to socio-contextual factors specific to the different study settings [17–20]. We considered multiple factors that might have resulted in more than 30 days patient delay [11,21]. In our study we found that many factors, i.e., age, TB symptoms, education, marital status, area of living, socio-economic characteristics, TB knowledge, and HSB were statistically significant in bivariate logistic regression. Surprisingly, very few participants (0.1%) mentioned they would go to pharmacies to seek treatment for TB, but actually 42.1% of them had visited their nearby pharmacy as first point of contact. After adjusting for covariates, the only factors that remained statistically significant were having diabetes, a bacteriological diagnosis and self-treatment. This varied from similar Asian and African studies, as they showed males, elderly age, lower education level and/or socioeconomic status, rural residence, stigma, lack of clinical symptoms, history of exposure to TB patients, undertaking multiple visits prior to diagnosis and initial visits to informal providers/traditional healers were significantly associated with patient delay [2,5,17–20,22]. Contrarily, some studies conducted in the Indian subcontinent and Iran also found that being ≥14 years old, female, urban residence, less knowledge regarding TB and having bacteriologically confirmed TB were associated with higher diagnostic and treatment delay [2,19,20,22]. Dramatic impact of the recent pandemic on TB services have also been reported by many studies and modelling estimates [23–26]. The pandemic affected PWTBs along with their communities, worsening existing challenges of TB programmes across the globe. Interestingly, our analysis showed that the male to female ratio had decreased to 1:1 during the strict lockdown period, possibly reflecting either increased healthcare awareness/HSB among females or reduced healthcare access among male participants. We also found that the duration of suffering and diagnostic delays had increased alarmingly after withdrawal of strict lockdown, implying many could not visit the health facilities during the lockdown period. This was confirmed by the fact that we had significantly less patients coming in from rural areas during the strict lockdown period.

Qualitative exploration documented the patients’ perception on the delays. Poor TB related knowledge, care seeking from informal providers during symptom onset and reluctance in seeking care from formal medical practitioners were found to be the key contributors. Such factors also persist in other LMICs. For instance, the effect of TB knowledge, attitude of healthcare providers and seeking treatment from non-medical health practitioners were reflected in a study from Sudan [27]. While most (80%) participants of this study had heard about TB, majority were not aware of the symptoms or transmission, thus relating to similar studies [28,29]. However, this contradicts the quantitative findings where participants demonstrated a strong knowledge regarding symptoms as well as knowledge on where to seek care from with those symptoms. One possible explanation is that people gained substantial knowledge about TB during their diagnostic journey, as during the qualitative exploration, they reflected on their poor TB knowledge at the time of symptom onset which they felt, was a major cause of delay in seeking care from appropriate facility. Our findings also indicated that participants preferred to seek initial care for cough, cold, and fever from informal providers and local pharmacies as the first point of contact, because of their availability, higher accessibility and low-cost [30]. They sought care from formal healthcare providers only after their conditions worsened, often contributing towards prolonged diagnostic delay, and increased risk of TB transmission to close contacts [31]. Socioeconomic condition also plays a key role in determining patients’ HSB and is a significant contributor towards delay. Patients often delay seeking formal care due to socioeconomic constraints, normalization of symptoms, and trust in informal or spiritual providers. As indicated in studies conducted in Ethiopia and Yemen and research on the political ecology of TB, we found that our participants were often from marginalised or disadvantaged groups [32–34]. Existing studies also present contrasting findings on association between LMIC status and patient delay. For instance, a qualitative study in Bangladesh found that underestimation of TB knowledge and lack of awareness among patients, along with opinions from family members, played key roles in their health-seeking behaviour [35]. Another study highlighted that the healthcare-seeking journey of extrapulmonary TB patients usually starts either at pharmacies or private facilities, with non-medical informants, mainly relatives and friends, influencing these decisions [36]. Since the beginning of the World Bank operations, Brazil has always been classified as an upper middle-income country, differing from other major emerging economies like China and India [37]. A study from Brazil found low income not to be associated with patient or healthcare delay [38]. One-third of participants (6/21) did not collect their diagnostic reports on time, and around a fourth delayed the initiation of treatment due to carelessness which is consistent with situations reported elsewhere [39]. Similar to reports from study participants in high and medium burden TB countries in Southeast Asia, our participants reportedly struggled to collect their TB medications on time due to their locations [40,41].

A limitation of our study was that many patients did not collect their reports themselves, or were severely ill and/or hospitalized. Hence, we might have missed a subgroup who have had late diagnosis or for whom, diagnosis of TB was incidental. Moreover, symptom onsets may not have been precisely described, as perceptions of illness or suffering may vary between individuals and over time. Recall bias was also a potential limitation among patients with long term illness.

The findings on patient delay and associated factors varied widely across studies and countries. Our participants were drawn from a public–private mix setup in Dhaka, the capital of Bangladesh. Majority were referred from private providers and hence the findings relate closely to the population seeking care in similar contexts. Addressing the challenges identified in this study will at the very least benefit the people from comparable settings, and potentially others.

## Conclusions

Delay between appearance of symptoms and initiation of TB treatment poses a major barrier in controlling the TB epidemic. Our analyses showed substantial diagnostic delays and highlighted that many participants lacked knowledge regarding the disease, its symptoms and where to seek care. This lack of awareness prompted their initial visit to informal providers and contributed towards the delay. These findings should be considered while planning for regular community awareness campaigns and improving access to TB care services. Future research should incorporate perspectives of healthcare providers to better understand how health professionals interpret and respond to these trajectories of pluralistic care-seeking. Organizing training for informal providers on TB basics and referral linkage, and connecting them with the national TB control program to integrate them into the TB care cascade, can help reducing health-system delays. Patient delay affects not only the individuals and their families, but also their overall community, and ultimately impacts the overall public health situation and economy of the country. It is thus imperative to take measures targeting delay reduction to lower TB incidence and meet the targets set in Sustainable Development Goals as well as fulfilling the objectives of the End TB Strategy.
