# Correction: Mixed-methods study to assess delay among patients with tuberculosis in an urban setting of Bangladesh

**DOI:** 10.1371/journal.pone.0337339

**Published:** 2025-11-19

**Authors:** The PLOS One Staff

The corresponding author is stated incorrectly as there is an Asterix(*) next to the wrong author’s name. The corresponding author is Sayera Banu.

The publisher apologizes for this error.
